# Recipes for Mindfulness in Your Library: Supporting Resilience and Community Engagement

**DOI:** 10.5195/jmla.2020.1076

**Published:** 2020-10-01

**Authors:** Kim K. Meeks

**Affiliations:** 1 meeks_k@mercer.edu, Skelton Medical Library, Mercer University School of Medicine, Macon, GA

*Recipes for Mindfulness in Your Library: Supporting Resilience and Community Engagement* offers insightful information not only for librarians, but also for anyone who is looking to provide mindfulness programming to groups or organizations and for those who want to find ways to improve themselves through mindfulness techniques. In the introduction, the editors—Madeline Charney, Jenny Colvin, and Richard Moniz—state that mindfulness should already be a personal practice for librarians as well as administrators who wish to implement these types of programs. The editors feel that the commitment to the personal practice of mindfulness from librarians and administrators brings authenticity to programs, which will help “this burgeoning movement take root with credibility and lasting value” (p xi).

The publication is a collection of stories provided by various contributors and gives anecdotal evidence of the efficacy of mindfulness techniques used in libraries. The book is neatly categorized into four parts. The first two sections look at programming and services relating to mindfulness, and the last two sections reflect on how librarians can utilize mindfulness practice to improve their relationships with coworkers and improve their teaching skills, while also sharing mindfulness skills with students in their classrooms.

The first section, “Library as Hub,” looks at mindfulness programming in a medical school, public library, academic library, and kindergarten–twelfth grade (K–12) public school system. This section shows readers how to create mindfulness programs and reviews different types of mindfulness techniques that have been used, for example, yoga and meditation. This section provides readers with what worked and what did not work in these programs. An interesting observation made in the first chapter of the book by authors Rebecca Snyder and Robin O'Hanlon was that mindfulness programs might be hard to reproduce because “in the spirit of contemplative practice…designing an intervention can be more of an art than a replicable scientific process,” (p. 7), and the design of a program in a different place might need to be totally different than the original. This leads readers to conclude that there is not a “one size fits all” approach and that to create a successful mindfulness program, the needs of users and the organization should be considered.

The second section of the book reviews innovative services that are being utilized in libraries. Many programming librarians will find this section immensely helpful, because it provides readers with unique ways to explore and conduct mindfulness programming. Many of the libraries in this section pushed boundaries and thought “outside of the box” on these projects. One of the chapters centers around McQuade Library at Merrimack College, which is a small college campus library that uses themed library kits for student use, including kits on bird watching, balancing chakras, gardening, and sound healing.

Another chapter discusses the Library Brain Booth from the Humboldt University Library, which was created for students with “the goal of introducing mindfulness as a means of introducing students to metacognition for academic success” (p. 37). According to the author, Katia G. Kardjova, the three techniques to accomplish this goal include intentional brain breaks, emotional self-regulation, and singular thoughtful focus (p. 38). This was a successful endeavor by the library and Kardojova, who went on to present the findings at various conferences.

University of West Florida's John C. Pace Library created a Library Zen Zone where students use Muse meditation headbands while listening to relaxing sounds. The headband connects to a sound application and allows users to see their brain activity while listening to these sounds. The goal of the Library Zen Zone is to give students the tools to understand how they physiologically respond to meditation, while also providing support to help combat perceived stress and giving students the knowledge to reflect on ways to improve their meditation practice (p. 46).

The third section focuses on librarians at the personal and professional level. This section would be a good starting point for librarians who are new to mindfulness. The chapters in this section discuss work journaling to improve teaching and how mindfulness can help to effectively manage teams. A key point in effectively managing teams is to allow library faculty to share their successes and failures in a safe environment with their colleagues. The author of chapter 12, Jenny Colvin, notes “mindfulness in the face of failure acknowledges that this happened. In a moment where everything is going wrong, mindfulness creates community. When sharing a failure, mindfulness allows us to go through it together rather than alone. The only way out is through” (p. 80).

The final section of the book assesses mindfulness in teaching and research as it relates to librarians and students. The section reviews the effects of assigning students a tree observation journal to increase their meditation skills while sitting with the tree, which creates an opportunity for the student to self-reflect and grow. The section also reviews how Lisa Melendez, the author and librarian at a community and technical college, received grant funding to provide programming for mindfulness as it relates to equity and inclusion. Additionally, another chapter covers how a university created a mindfulness workshop to help library faculty reduce stress during teaching.

The publication is a fairly quick read, but it provides a wealth of information on many different levels relating to mindfulness. A list of contributors includes a biography and the qualifications of each. Librarians from various schools are represented, such as community colleges, technical colleges, university libraries, and public libraries. Many of the contributors are former or current librarians and library administrators. A thorough index is provided at the back of the book. I highly recommend this book to anyone who is looking for a solid overview on mindfulness as it relates to library programming and a better understanding of its effective use from a personal and professional standpoint.

